# Transgenic Mice Overexpressing PG1 Display Corneal Opacity and Severe Inflammation in the Eye

**DOI:** 10.3390/ijms22041586

**Published:** 2021-02-04

**Authors:** Min-Kyeung Choi, Minh Thong Le, Hye-Sun Cho, Juyoung Lee, Hyoim Jeon, Se-Yeoun Cha, Manheum Na, Taehoon Chun, Jin-Hoi Kim, Hyuk Song, Chankyu Park

**Affiliations:** 1 Department of Stem Cell Biology and Regenerative Biology, Konkuk University, Hwayang-dong, Seoul 05029, Korea; antares1029@gmail.com (M.-K.C.); minhthongbio@gmail.com (M.T.L.); chssky77@konkuk.ac.kr (H.-S.C.); holyboy91@nate.com (J.L.); kamuijhi@konkuk.ac.kr (H.J.); jhkim541@konkuk.ac.kr (J.-H.K.); songh@konkuk.ac.kr (H.S.); 2 College of Veterinary Medicine, Chonbuk National University, Iksan 54596, Korea; seyeouncha@gmail.com; 3 Department of Biotechnology, College of Life Sciences and Biotechnology, Korea University, Seoul 02841, Korea; nmhsoul@gmail.com (M.N.); tchun@korea.ac.kr (T.C.)

**Keywords:** protegrin, antimicrobial peptide, cathelicidin, transgenic mice, corneal opacity

## Abstract

Antimicrobial peptides (AMPs) are of interest as alternatives to antibiotics or immunomodulators. We generated and characterized the phenotypes of transgenic mice overexpressing protegrin 1 (PG1), a potent porcine cathelicidin. No obvious differences were observed between PG1 transgenic and wild-type mice in terms of growth, development, general behaviour, and the major immune cell population. However, PG1 transgenic mice intranasally infected with *Staphylococcus aureus* resulted in a reduction in microscopic pulmonary injury, improved clearance of bacteria, and lower proinflammatory cytokine secretion, compared to those of wild-type mice. On the other hand, approximately 25% of PG1 transgenic mice (*n* = 54/215) showed corneal opacity and developed inflammation in the eye, resulting ultimately in phthisis bulbi. Immunohistochemical analyses revealed that PG1 and its activator, neutrophil elastase, localized to the basal cells of the cornea and glands in eyelids, respectively. In addition, apoptosis indicated by a Terminal deoxynucleotidyl transferase dUTP nick end labelling (TUNEL)-positive signal was detected from flat cells of the cornea. Our study suggests that the expression regulation or localization of AMPs such as PG1 is important to prevent their adverse effects. However, our results also showed that the cytotoxic effects of PG1 on cells could be tolerated in animals, except for the eyes.

## 1. Introduction

Cathelicidins are a family of antimicrobial peptides that contain a highly conserved cathelin domain in the prepropeptide form and diverse cationic mature-peptide regions [1,2]. Cathelicidins are stored in granules within neutrophils as inactive propeptides [3]. Elastase can cleave the cathelin domain of the propeptides and convert them to mature PG1 (mPG1), the active form that exerts potent and broad spectrum antimicrobial activities against bacteria [3,4], fungi [5], and viruses [6,7] and exerts cytotoxic effects against cancer cells [8]. Cathelicidins also function as immune modulators in leukocyte activation, immune cell recruiting, wound healing, inflammation suppression, and lymphocyte differentiation [2,9,10,11].

Cathelicidin deficiency has been reported to reduce wound healing capacity after Streptococcal infection and severe periodontal disease [12]. However, overexpression of LL-37 was also reported to be associated with psoriasis [13,14], suggesting that the regulation of cathelicidin expression is important. Protegrins (PGs) comprise a group of cathelicidins in pigs that were originally identified from swine leukocytes [15]. However, our recent study showed that several previously reported PGs were different allelic forms derived from a single locus [16]. PGs exhibit typical characteristics of antimicrobial peptides, including small size, cationic and amphipathic topology, and broad antimicrobial spectrum [17,18,19,20,21]. In contrast to salt-sensitive defensins, PGs are relatively resistant to changes in NaCl concentration [22].

There has been increasing research interest in the use of antimicrobial peptides (AMPs), including cathelicidins, as promising drug candidates that can serve as substitutes or complementary medication to conventional antibiotics [23]. However, the systemic effects of AMPs as pharmaceutical substances including cytotoxicity require further study. Rapid presystemic elimination of AMPs by endogenous proteinases may render them ineffective when administered directly. AMPs may also be cytotoxic to a part of mammalian systems [24,25]. Therefore, stable in vivo animal model experiments are necessary and powerful to study the systemic effect of AMPs.

Mucins are expressed at the apical surface of epithelial cells in the lungs, stomach, intestines, eyes and several other organs and serve as the first line of defence for epithelial tissues against pathogenic infection [26]. Here, we produced PG1-overexpressing transgenic mice under the control of the porcine *MUC1* promoter and analysed the corresponding systemic effects on animal development and immune cells. Our study suggests that the expression regulation or localisation of AMPs such as PG1 is important to prevent their adverse effects.

## 2. Results

### 2.1. Generation of PG1-Overexpressing Transgenic Mice

We chose the promoter of the porcine *MUC1*, a membrane-anchored mucin, for *PG1* expression to restrict the expression of the transgene mainly to mucosal membranes, where innate immune defence could be more critical than other tissues while considering possible applications in pigs [27]. First, we attempted to prepare the preproPG1 (pPG1) overexpression construct under the control of the putative porcine mucin 1 (*MUC1*) promoter. The 150-bp *MUC1* promoter sequence, which exhibits high sequence similarity (77.22%) with the upstream 2-kbp regions from the start codon of *MUC1* in mice and pigs, was used as the putative *PG1* promoter (Figure S1). The pMUCPG1 PG1 expression construct was generated by ligating PCR amplicons of the putative *MUC1* promoter sequence to the pCAGGS mammalian expression vector containing the 447-bp full-length cDNA sequence of *PG1* (Figure S2). Subsequently, the construct was linearized and used in pronuclear injections to produce PG1 transgenic mice. A total of 18 PG1 transgenic founder animals (nine males, nine females) were obtained. Correct integration of the transgene was confirmed based on the presence of 465-bp *PG1*-specific amplicons obtained from genomic PCR using the primers pPG1-F and -R (Figure S3).

### 2.2. PG1 Expression Patterns under the Control of the Putative Porcine MUC1 Promoter

To analyse the expression patterns of *PG1* in the transgenic mice, total RNA was isolated from 14 different tissues of the transgenic mice, after which *PG1* expression levels were determined using semi-quantitative reverse transcription (RT) PCR (Figure S4A). The eye, kidney, stomach, spleen, testis, uterus, and muscle showed higher *PG1* expression levels compared to other tissues. *PG1* had moderate expression in the neocortex, lung, and ovary but had low expression in the thymus and skin. The observed *PG1* expression patterns in the transgenic mice were consistent with that of the murine *Muc1* gene reported in a public gene expression database (GeneAtlas MOE430), which reported strong *Muc1* expression in the lung, uterus, ovary, stomach, cornea, and testis, although the list of tissues was not identical to those used in our experiment. Our results indicate that *PG1* expression under the control of the putative porcine *MUC1* promoter resembles the natural expression patterns of endogenous *Muc1* in mice. In addition, *PG1* was also expressed in peripheral blood mononuclear cells (PBMCs) of PG1 transgenic mice (Figure S4B).

### 2.3. Analysis of Immune Cell Populations in PG1 Transgenic Mice

Antimicrobial peptides could influence the immune system by acting as immune modulators [28,29]. To explore the possibility of using PG1 as an immune modulator, we evaluated the changes in population sizes of major immune cells in PG1 transgenic mice and compared the results against wild-type mice. Population sizes of T-cells (TCR β^+^), regulatory T-cells (CD4^+^ and CD25^+^), B-cells (B220^+^), granulocytes (Gr1^+^), and antigen presenting cells, including macrophages (CD11b^+^) and dendritic cells (CD11c^+^), were analysed via fluorescence-activated cell sorting (FACS). FACS analysis was carried out on the spleen and mesenteric lymph nodes of PG1 transgenic mice (Table 1 and Table S1), as well as natural killer T cell (NKT) cells (TCRβ^+^ and NK1.1^+^) obtained from the spleen. However, no significant differences in population sizes were observed between wild-type C57BL/6 and PG1 transgenic mice. In addition, there were no differences in terms of the numbers of activated CD4^+^ (CD4^+^, CD44^+^, CD69^+^, and CD62L^−^) and CD8^+^ (CD8^+^, CD44^+^, CD69^+^, and CD62L^−^) T-cells in the spleen and mesenteric lymph nodes, suggesting that PG1 does not play a significant role as an immune cell activator at least in this study.

### 2.4. Enhanced Respiratory Resistance to Experimental Intranasal Infection with Staphylococcus aureus in PG1 Transgenic Mice

**Figure 1:**
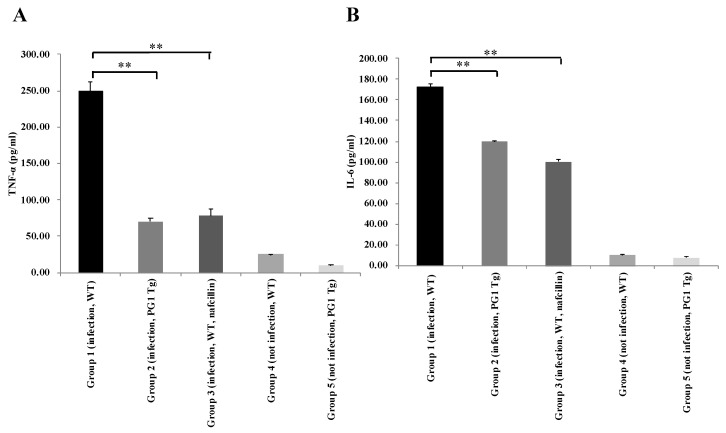
Comparison of the level of TNF-α and IL-6 secretion in the lung between wild-type and PG1 transgenic mice 48 h after *Staphylococcus aureus* infection. Except for groups 4 and 5, all other groups were challenged intranasally with *S. aureus.* The measured levels of TNF-α (**A**) and IL-6 (**B**) using ELISA assays were indicated. The symbol ** indicates *p*-value < 0.01. WT, wild-type; Tg, transgenic; Group 4, the control group.

We evaluated the effect of ectopically expressed PG1 against intranasal inoculation of *S. aureus* using PG1 transgenic mice. PG1 expression in the lung was confirmed by immunohistochemistry (Figure S5C). Five different experimental groups depending on the presence (+) and absence (-) of bacterial infection, PG1 transgene and nafcillin treatment were analysed (Table S2). The lungs were collected at 4, 12, 24 and 48 h after inoculation. The gross appearance and microscopic analysis of haematoxylin and eosin (H&E) stained tissue sections among different groups showed that the pulmonary injury, such as acute interstitial and alveolar oedema and patchy haemorrhage, was significantly reduced in both groups 2 (+/+/-, bacterial infection, PG1 transgenic and no nafcillin treatment, *n* = 12) and 3 (+/-/+, *n* = 10) (Figures S6 and S7). The bacterial load at the lung 24 and 48 h after infection was also significantly lower in groups 2 and 3 than group 1 (+/-/-, *n* = 12, Figure S8). Immunohistochemical analysis showed a reduction in the infiltration of immune cells from groups 2 and 3 over group 1 (Figures S7 and S9). The level of pro-inflammatory TNF-α and IL-6 was also significantly lower in groups 2 and 3 than group 1 (*p*-value < 0.01, Figure 1). This could be due to the fact that infection is resolving in PG1 transgenic mice rather than PG1 having any direct effect on inflammation. There was no difference in the results of histological analysis and the level of TNF-α and IL-6 between wild-type and PG1 transgenic lungs.

### 2.5. Corneal Opacity and Subsequent Inflammation in the Eyes of PG1 Transgenic Mice

Multiple PG1-transgenic founders were generated. Initially, no phenotypic differences were identified between transgenic and wild-type litter mates in terms of development, growth, and general behaviour. In addition, the phenotypes of different PG1 transgenic lines did not exhibit observable differences. Interestingly, however, a high frequency of corneal opacity was observed in PG1 transgenic mice compared to that of wild-type mice and to previous studies reporting only 4.1% corneal opacity in C57BL/6 mice and a very low incidence of corneal opacity (1.6%) in gnotobiotic mice [30]. Similar results were also observed from other PG1 transgenic lines. A total of 25.1% (*n* = 54) of PG1 transgenic mice presented corneal opacity in either one or both eyes at 6 months of age (Table 2 and Figure S10). However, 74.9% (*n* = 161) of the transgenic mice did not develop corneal opacity until 6 months of age, demonstrating incomplete penetrance of the phenotype.

**Figure 2:**
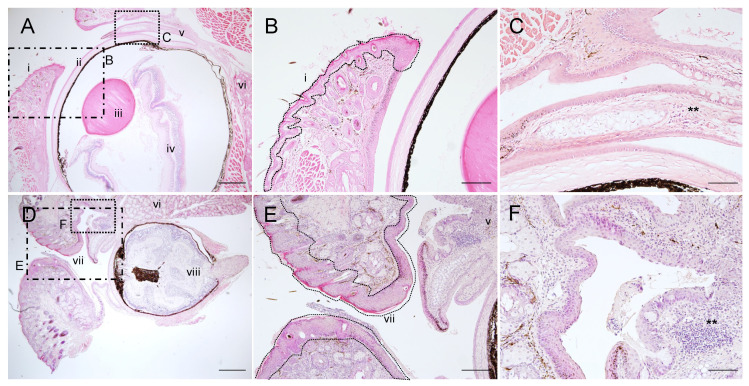
Representative images of inflammation in the eyelid and subsequent eye deformation in PG1 transgenic mice. Haematoxylin and eosin (H&E) staining of the eyelid and ocular regions from wild-type (**A**–**C**), abnormal PG1-TG (**D**–**F**). (i) eyelid in normal condition, (ii) cornea, (iii) lens, (iv) retina, (v) conjunctiva, (vi) Harderian gland, (vii) thickened eyelid epithelium, (viii) shrinking eyeball. The rectangular insets in figures (**A**,**D**) were enlarged in (**B**,**C**,**E**,**F**). The symbol ** in figure F indicates a cluster of infiltrated granulocyte-like cells. Scale bar represents 500 μm (**A**,**D**), 200 μm (**B**,**E**), and 50 μm (**C**,**F**), respectively.

Corneal opacity was detected as early as 3 weeks and became apparent at 3 months in all affected mice (Figure S10). Once inflammation occurs (Figure S10B), the upper and lower eyelids attach gradually and eventually block the eye opening, although the severity of the symptoms shows individual variation. The eyeballs in affected eyes were also observed to degenerate over time and were shrunken and non-functional, which is the typical phenotype of phthisis bulbi in humans (Figure 2D and Figure S11G,H). Moreover, significantly higher numbers of infiltrated granulocyte-like or eosinophilic immune cells were present in PG1 transgenic ocular tissues (Figure 2C,D and Figure S11B,D,F).

### 2.6. Immunohistochemical Analyses of PG1 Expression in the Eyes of PG1 Transgenic Mice

**Figure 3:**
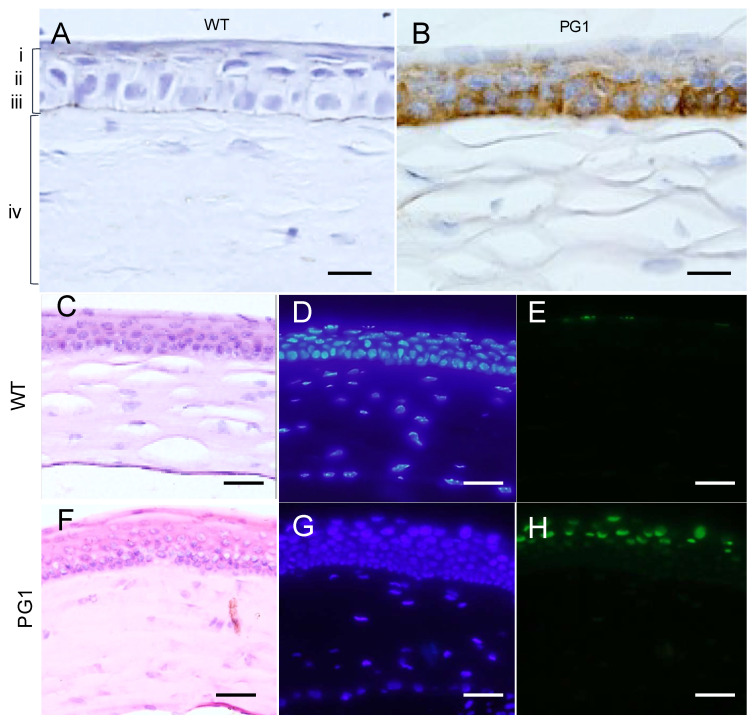
Results of immunohistochemical analyses using anti-PG1 antibodies and TUNEL assay against the cornea of PG1 transgenic mice affected with corneal opacity. (**A**–**H**) represent the results of anti-PG1 immunohistochemistry, H&E staining, DAPI staining, and TUNEL analysis, respectively. Corneal epithelium cells are marked as (i) squamous, (ii) wing, (iii) basal, (iv) stroma. (**A**,**C**–**E**) are from wild-type (C57BL/6) mice, while (**B**,**F**,**G**,**H**) are from PG1 transgenic mice. Basal cells of the cornea shown in brown were reactive with anti-PG1 antibodies by diaminobenzidine (DAB) staining (**B**). The anti-PG1 signals were similar between affected and not affected PG1 transgenic mice. TUNEL-positive signals were detected from the flat cells of the cornea of the transgenic mice (**H**). Opacity-affected eyes show thickened epithelia (**C**) vs. (**F**). Scale bars represent 20 μm and 50 μm. for (**A**,**B**) and (**C**–**H**), respectively.

To determine the specific cell type expression and distribution of PG1 in the eyes of PG1 transgenic mice, we carried out immunohistochemical analyses on eye sections using anti-PG1 antibodies. Positive signals were observed from the basal cells of cornea of the PG1 transgenic mice (Figure 3A,B). In addition, the affected corneal epithelia of PG1 transgenic mice were thicker compared to the wild-type owing to cell proliferation and migration (Figure 3F–H). Except for the corneal region, continuous PG1 expression was not detected in circumocular tissues. TUNEL analysis was also carried out to detect cells undergoing programmed cell death as a consequence of PG1 expression in the eyes. Positive signals for programmed cell death were observed from the flat cells but not from basal cells of the cornea (Figure 3D,E,G,H). In addition, punctate *PG1* expression was detected in the retina, especially in the outer plexiform layer (Figure S5A,B). Taken together, these results indicate that PG1 expression patterns in transgenic mice account for the eye abnormalities observed in PG1 transgenic mice.

### 2.7. PG1 Treatment on Eyes Results in Corneal Opacity

To validate our hypothesis that PG1 expression is the direct cause of corneal opacity, the eyes of wild-type C57BL/6 mice were treated with an eye drop solution containing chemically-synthesised PG1 peptides at a concentration of 12 µM, which corresponds to the half maximal effective concentration (EC_50_) for PG1 against the human erythroleukemia K562 cell line [31]. Because EC_50_ of PG1 to corneal cells in mice was not available, we alternatively used the reported EC_50_ value of a mammalian cell line, K562. After 5 consecutive days of PG1 treatment every 12 h, all animals (*n* = 3) in the PG1-treated group exhibited corneal opacity (Figure S12). Thus, the results support the relationship between corneal opacity and the ectopic expression of PG1 in transgenic mice.

### 2.8. Comparison of Cytotoxicity between Pro- and Mature Peptides of PG1 in Mammalian Cells

PreproPG1 (pPG1) is known to be relatively inactive and non-toxic to mammalian cells, whereas mature PG1 (mPG1) is active and cytotoxic [25]. Therefore, the occurrence of corneal opacity in PG1 transgenic mice is likely to involve the activation of preproPG1 in the eyes. Neutrophil elastase was reported to induce cleavage of prepropeptides to the mature form in mammalian cells and is known to be mainly produced by neutrophils [25]. However, the eyes are immune-privileged organs that limit the entry of immune cells [32]. Therefore, the exact mechanisms explaining the presence of PG1 in eyes, where neutrophils are not present, were unclear. In the eyes of PG1 transgenic mice, neutrophil elastase from the sebaceous gland of eye lids and Harderian glands may contribute to converting preproPG1 on the corneal surface to the active form (Figures S6 and S14). Our analysis showed the expression of PG1 in the corneal epithelium and outer plexiform layer of PG1 transgenic eyes (Figure 3 and Figure S5). Thus, we used epithelial fibroblast and retinal cell lines for further in vitro analyses.

**Figure 4:**
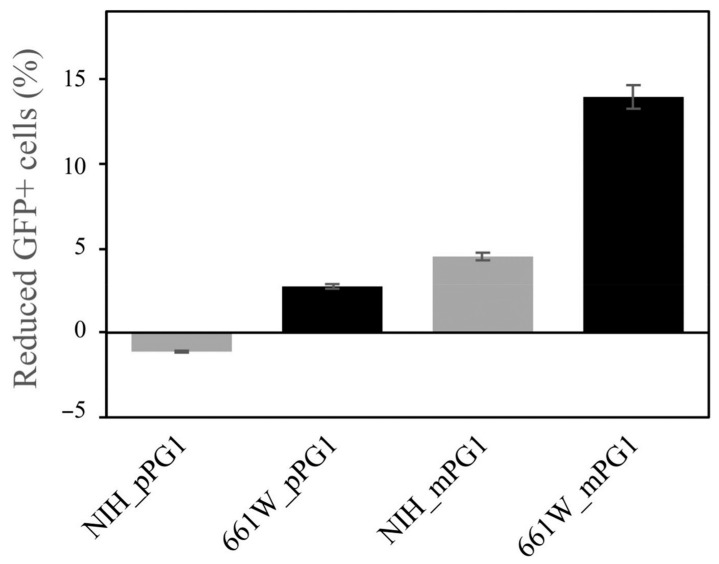
Changes in the viability of cells after PG1 expression. EGFP-tagged preproPG1 (pPG1) and mature PG1 (mPG1) were separately transfected into NIH3T3 and 661W cells. The number of EGFP-positive cells was measured 16 h after transfection. *mPG1*-transfected cells showed a significant decrease in the number of EGFP-positive cells compared to that of pPG1.

**Figure 5:**
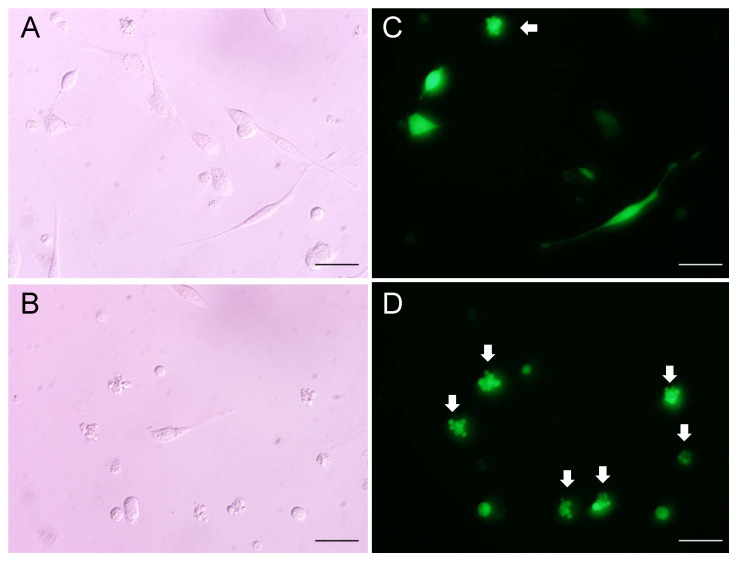
Analysis of cell death after mPG1 transfection using light and fluorescence microscopy. EGFP signals were compared between NIH3T3 (**A**,**C**) and 661W (**B**,**D**) cells after transfection with EGFP-mPG1. Arrows indicate cells undergoing cell death based on cell fragmentation. Scale bars represent 50 μm.

To verify whether the abnormal eye phenotypes in PG1 transgenic mice can be directly caused by pPG1, we transfected a fibroblast cell line, NIH3T3, and a retinal neuronal cell line, 661W, with EGFP-tagged mPG1 (EGFP-mPG1) and pPG1 (EGFP-pPG1), respectively (Figure S13). RT-PCR results confirmed that murine neutrophil elastase mRNA is not expressed in both cells. At 16 and 24 h after transfection, counts of EGFP^+^ cells were estimated for each time point and used as indicators of cell viability. *EGFP-pPG1*-transfected NIH3T3 and 661W cells did not show significant differences in viability compared to controls transfected with EGFP alone (Figure 4). By contrast, EGFP^+^ cell counts in *mPG1*-transfected 661w and NIH3T3 cells decreased by 13.94% and 4.50, respectively, at 16 h after transfection, indicating that mPG1 significantly influences the survival of transfected cells. Microscopic observations revealed a large number of dying EGFP^+^ cells displaying fragmented morphology (Figure 5). Our results also indicate that 661w cells, which originate from retinal neurons, are more susceptible to the cytotoxic effects of PG1 than NIH3T3 cells with fibroblast lineage, thereby demonstrating the differences in mPG1 sensitivity among different cell types. This is consistent with the results of PG1 cytotoxicity towards mammalian cells evaluated using the MTT assay [24]. These results could explain the abnormality in eye tissues, but require the conversion of pPG1 to mPG1.

### 2.9. Expression of Murine Neutrophil Elastase (ELANE) in the Eyes

**Figure 6:**
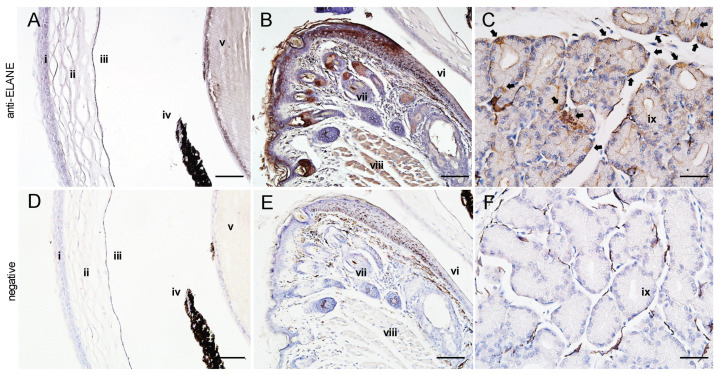
Immunohistochemical analysis of the cornea and eyelid of PG1 transgenic mice using anti-ELANE antibodies. (**A**–**F**) are the results of anti-ELANE immunohistochemistry for the corneal region, eyelid, and Harderian gland, respectively. Antibody reactivity is indicated in brown by diaminobenzidine staining. Sebaceous glands in the eye lids ((**B**), 200×) and type-I cell-like cells in Harderian glands ((**C**), 400×, black arrows indicated stained cells) showed positive signals for ELANE. The staining pattern of wild-type mice with anti-ELANE was identical to that of PG1 transgenic mice. (**A**–**C**): anti-ELANE antibodies. (**D**–**F**): negative controls. (i) corneal epithelium, (ii) corneal stroma, (iii) corneal endothelium, (iv) iris, (v) lens, (vi) palpebral conjunctiva, (vii) hair follicle, (viii) orbicularis oculi muscle, (ix) Harderian gland. Scale bars represent 100 μm.

Neutrophil elastase cleaves the loop region between the anionic cathelin domain and the cationic mPG1 region to produce the functionally active peptide, mPG1 [25]. To identify the sources of the PG1-activating enzyme in the eyes of transgenic mice, we performed immunohistochemical analyses on the eye sections using anti-murine neutrophil elastase (ELANE) antibodies. The corneal region did not show ELANE expression (Figure 6A). However, the sebaceous gland (Meibomian and Zeis glands), which secretes lipids to the superficial layer of the tear film in eyelids, and type-I cell-like cells in the Harderian gland, showed ELANE antibody-specific signals (Figure 6B,C and Figure S14). These results demonstrate the expression of ELANE or ELANE-like enzymes specific to anti-ELANE antibodies in the eyes, and therefore suggest that ELANE is expressed in other cell types aside from neutrophils.

## 3. Discussion

Antimicrobial peptides with potent and broad spectrum antimicrobial activities have increasingly drawn attention as alternatives to conventional antibiotics [33]. However, in addition to their beneficial effects, understanding of the cellular toxicities and corresponding molecular mechanisms associated with AMPs requires further investigation for their use in biomedical applications. Several studies have reported unexpected consequences of AMP expression in addition to their antimicrobial properties [31,34]. In this study, the ectopic expression of PG1 resulted in frequent occurrences of corneal opacity and ultimately phthisis bulbi. It is known that phthisis bulbi occurs from severe eye disease, inflammation or injury, or it may represent a complication of eye surgery [35,36,37]. Therefore, the corneal opacity in PG1 transgenic mice is likely to be the first external sign of the underlying lesion from inappropriate PG1 expression in the eyes.

The most common causes of corneal opacities in humans include old smallpox, trachoma, and keratomalacia [38], whereas minor causes include chemical and mechanical injuries. Changes in the ultrastructure of the cornea induced by inflammation, swelling, postsurgical, and wound healing can lead to loss of transparency of the cornea [39].

Our study detected strong cell death signals in the corneal epithelium of PG1 transgenic mice (Figure 3). Corneal cell death and the subsequent wound healing process are likely to be the main causes of corneal opacity in the transgenic mice. PG1 has been suggested to form ion channel-like pores in the cell membrane that could cause efflux or influx of ions, which are crucial for biological activities and membrane depolarisation [40,41]. In turn, these changes may induce progressive corneal cell death and subsequent accumulation of scars, cell migrations, and proliferation of corneal epithelium cells during the repair process.

A previous study reported that PG1 overexpression under the control of the cytomegalovirus (CMV) promoter improved resistance against Gram-negative bacterial infection in the murine respiratory tract [42]. The study reported increased neutrophil recruitment at infected sites in transgenic mice. However, definite conclusions cannot be drawn based on the results of the pathogenic challenge experiment in our PG1 transgenic model. This could be due to various reasons, including differences in the PG1 expression patterns between the two studies.

Environmental cues such as pathogens, allergens, and dust particles can activate immune systems and recruit immune cells to the affected region [43]. The recruitment of neutrophils is especially important for cathelicidins such as PG1 to convert the inactive form (prepro PG1) to the active form (mature PG1). Defects in immune cells should contribute to failure in lymphocyte recruitment including neutrophils [44]. However, we were not able to find any evidence of immune defect phenotypes from our PG1 transgenic mice even under the conventional environment. Further investigation is necessary to determine the differences in the in vivo effects of PG1 expression.

Comparative analysis of immune cell populations between wild-type and PG1 transgenic mice did not show clear differences in immune cell populations, indicating that the effect of PG1 could be limited to direct interactions with the cell membrane to cause membrane disruption. The image of cell fragmentation in Figure 5 observed from the *EGFP-mPG1* transfection experiment is also consistent with observed membrane disruption by PG1. In addition, our results suggest that immune cells are less susceptible to the direct toxicity of PG1. Several in-vitro studies reported the possible immunomodulatory effect of AMPs [28,29]. However, supporting evidence from in vivo studies is currently not available, and we were unable to detect any systemic changes in population sizes of major immune cells from the analysis of our PG1 transgenic mouse model (Table 1). A high-level expression of *PG1* in the spleen and the confirmation of *PG1* expression from purified PBMCs in our transgenic mice (Figure S4B) suggest the possible exposure of immune cells to PG1. However, we were not able to validate the amount of active PG1 converted from preproPG1. A fraction of active PG1 could be generated and interact with immune cells considering the occurrence of eye phenotypes in the transgenic mice. Although PG1 can potentially serve as a chemoattractant for lymphocytes or induce ELANE [42], the result in this study did not support such a conclusion. Further studies on the role of AMPs as possible immune modulators, especially with in-vivo models, are required to further investigate the immune modulatory function of AMPs and to draw a more definite conclusion on this issue.

AMPs display a broad spectrum of antimicrobial activities. In addition, several studies also demonstrated the cytotoxic effects of AMPs on mammalian cells. For example, LL-37 showed cytotoxicity against both prokaryotic and eukaryotic cells [45]. In addition, gomesin, tachyplesin, and polyphemusin II, as well as their linear analogues, were shown to be capable of inducing cell death in mammalian cells (K562 cells) [31]. Overexpression of beta-defensin 6 was shown to result in muscle degeneration [34]. By contrast, our results show that in vivo cellular toxicity or abnormal effects of PG1 could be tolerated in animals except for the eyes, thereby highlighting the therapeutic potential of AMPs such as cathelicidins. For future applications, structural modification can be employed to decrease or eliminate the cytotoxic effects of AMPs on mammalian cells. For example, the haemolytic activity of cyclised PG1 against mammalian cells was demonstrated to be tenfold lower compared to linear PG1 and did not lead to a reduction in inherent antimicrobial activity [46].

PG1 was expressed in several tissues in the transgenic mice, but the phenotypic abnormality was only observed in the eyes. This could be explained by differences in sensitivity of epithelial (NIH3T3) and retinal (661W) cells against PG1 toxicity (Figure 4 and Figure 5). The cellular toxicity of PG1 has been reported to vary depending on cell type [24,31]. For example, differences in haemolytic effects of PG1 between human and goat or sheep red blood cells were suggested to be caused by differences in lipid composition [21,47].

The underlying mechanisms behind protegrin activity were proposed to be membrane disruption and subsequent induction of free radical and Ca^2+^ influx, which promotes death in mammalian cells [31]. In particular, Ca^2+^ influx induces excitotoxicity in neurons by excessive depolarisation of the postsynaptic membrane [48]. This is consistent with our results showing that *PG1*-transfected 661W neuronal cells showed higher PG1 sensitivity than NIH3T3 fibroblast cells. Although 661W is an immortalized cone photoreceptor cell line derived from the retinal tumour of a mouse expressing SV40 T antigen [49], 661W cells express the majority of markers of cone cell origin and grow long primary cilia, indicating that these cells can be used as an in vitro photoreceptor model considering difficulties with the purification and genetic manipulation of photoreceptor cells [50]. Not all PG1 transgenic mice developed corneal opacity. Variations in phenotypic expression under the control of the same effector gene or mutation can occur because of differences in genetic background or the presence of modifying genes [51]. However, the PG1 transgenic mice used in the experiments share an identical genetic background. Furthermore, variation in phenotype expression was consistently observed from multiple independent PG1 transgenic lines and regardless of living conditions, specific pathogen free (SPF) and conventional environments. Thus, a random and unknown cellular stochastic event involving PG1 or environmental effects may be responsible for the observed differences in phenotypic expression.

In this study, we showed that the cytotoxic effect of PG1 could cause severe ocular lesion in animals while it could be tolerated in other parts of the body. Interestingly, the PG1 transgenic mice could be used as a model for ophthalmic research on phthisis bulbi. Our results also indicate that appropriate peptide modifications or exposure control are necessary to avoid the cytotoxic effects of natural AMPs on mammalian cells.

## 4. Materials and Methods

### 4.1. Preparation of the PG1 Expression Construct

The porcine *Muc1* promoter sequence, located 1–150 bp upstream of the start codon (Figure S1), was amplified from pig genomic DNA using primers SalI-MUCp-F (5′-GTC-GAC-GGC-GGA-GCT-CTG-TCA-CCT-3′) and (MUCp-ApaI-R: 5′-GGG-CCC-GAT-GGC-GGT-AAA-GTG-GTG-GG-3′). The amplicon was digested with *SalI* and *ApaI* (NEB, Ipswich, MA, USA) to generate a 160 bp region containing the enzyme recognition sequence and was purified from agarose gel using QIAquick Gel Extraction Kit (Qiagen Science Inc., Germantown, MD, USA) according to manufacturer instructions. Purified fragments were ligated into the modified mammalian expression vector pCAGGS following the removal of the original vector’s enhancer and promoter regions [52]. To obtain the intact *PG1* coding sequence, total RNA was isolated from pig peripheral blood mononuclear cells (PBMCs) using TRIzol reagent (Invitrogen, Carlsbad, CA, USA) according to manufacturer instructions. Isolated RNA was subjected to RNase-free-DNase I (Qiagen Science Inc., Germantown, MD, USA) treatment. RNA quality was analysed on a 2% formaldehyde agarose gel. Reverse transcription (RT) was performed in 20 μL reaction volumes using 3 μg of total RNA with oligo-(dT)_15_ primers and SuperScript^®^ III Reverse Transcriptase (Invitrogen, Carlsbad, CA, USA). Incubation was carried out for 50 min at 50 °C, followed by inactivation for 15 min at 72 °C. Subsequently, PG1 cDNA was amplified using 2 μL of RT products in reactions containing the primers pPG-F (5′-GCT-CTA-GAA-CCA-TGG-AGA-CCC-A-3′ and pPG–R (5′-TCC-TCG-AGT-CAT-CCT-CGT-CCG-A-3′), proofreading DNA polymerase (Pyrobest^TM^, TAKARA, Tokyo, Japan), buffer, and dNTPs (TAKARA, Kusatsu, Shinga, Japan). PCR conditions were as follows: 5 min denaturation at 94 °C; 30 cycles of 30 s denaturation at 94 °C; 30 s annealing at 60 °C; 30 s extension at 72 °C; and 5 min final extension at 72 °C. *XbaI*- and *XhoI*- (NEB, Ipswich, MA, USA) digested cDNA was ligated into the modified pCAGGS vector with T4 DNA ligase (TAKARA, Kusatsu, Shinga, Japan) (Figure S2).

### 4.2. Generation of Transgenic Mice

The transgenic construct (pMUCPG1, Figure S2) was digested with *SalI* and *PstI*. The 1925 bp fragment containing the *MUC1* promoter and *PG1* cDNA was isolated and gel-purified as described above. The purified DNA was microinjected into the male pronucleus of the fertilised eggs of C57BL/6 mice. Eggs were implanted into the oviducts of pseudo-pregnant ICR recipients using a commercial service (Macrogen, Seoul, South Korea). To confirm genomic integration of the transgene, tail lysates of new-born offspring were subjected to PCR in 15 μL reaction volumes using the primers pPG-F and –R as described above. Tail lysates were prepared using DirectPCR Lysis Reagent (Viagen Biotech, Los Angeles, CA, USA) according to manufacturer protocols. Multiple PG1-transgenic founders were generated, and each founder was bred separately. A PG1 transgenic line was selected and used for all phenotypic analyses. Animal care was approved and supervised by the Institute of Animal Care and Use Committee of Konkuk University (KU15119). Mice were maintained under specific pathogen-free conditions with unrestricted feeding and housed in groups of five mice per cage. All procedures and experimental protocols involving mice followed the Guidelines for Accommodation and Care of Animals (European Convention for the Protection of Vertebrate Animals Used for Experimental and Other Scientific Purposes) and internal guidelines. All tissue samples were collected after euthanasia in a CO_2_ chamber.

### 4.3. RNA Isolation and Semi-Quantitative RT-PCR

Tissues were dissected, snap frozen in liquid nitrogen, and stored at −80 °C until use. PBMCs were isolated from heparinized blood using the standard ficoll gradient method [53]. Total RNA was extracted from frozen tissues using TRIzol reagent (Invitrogen, Carlsbad, CA, USA) according to manufacturer protocols. cDNAs were synthesised using the method described above. Target cDNAs were amplified using 2 μL of RT products following the same procedure as the genomic PCR described above but using the primers PGRT-F (5′-CCT-CGG-AAG-CTA-ATC-TCT-AC-3′) and PGRT-R (5′-GAC-ACA-GAC-GCA-GAA-CCT-AC-3′) for *PG1* and ELANERT-F (5′-CTA-CTG-GCA-TTG-TTC-CTG-3′) and ELANERT-R: (5′-CAG-ACA-GGT-CCT-AGT-TGG-T-3′) for *ELANE*. Thermal cycling conditions were as follows: 5 min denaturation at 94 °C; 30 cycles of 30 s denaturation at 94 °C; 30 s annealing at 60 °C; 30 s extension at 72 °C; and final extension for 5 min at 72 °C for *PG1*. For *ELANE*, conditions were the same as that of *PG1* but with a 50 s extension period. *GAPDH* was used as an internal standard for gene expression analysis. Primer sequences for *GAPDH* were 5′-GCT-ACA-CTG-AGG-ACC-AGG-TTG-3′ and 5′-AGG-AGA-TGC-TCG-GTG-TGT-TG-3′. RT-PCR reactions were performed at least thrice. PCR products were run on 1.5% agarose gels, stained with ethidium bromide, and visualised with UV illumination. *PG1* mRNA expression levels were estimated according to photo-density ratios between the amplicons and *GAPDH* using ImageJ (NIH, Bethesda, MD, USA).

### 4.4. Immunohistochemical Analysis

Tissues were incubated in 4% paraformaldehyde solution overnight, dehydrated, and embedded in paraffin. Tissues were cut into 5 to 7 μm thick sections using a microtome (Leica, Wetzlar, Germany) and mounted onto poly-L-lysine-coated glass slides. After deparaffinisation and hydration, sections were incubated in 3% hydrogen peroxide for 30 min and incubated with rabbit anti-PG1 [16] and anti-ELANE (1:400 dilutions, Santa Cruz Biotechnology, Dallas, TA, USA) antibodies in phosphate-buffered saline (PBS) containing 5% normal sheep serum and 0.5% Triton-X (Sigma-Aldrich, St. Louis, MO, USA). Standard avidin-biotin immunohistochemistry was performed using a rabbit ExtrAvidin^®^ Peroxidase Staining Kit (Sigma-Aldrich, St. Louis, MO, USA) according to manufacturer protocols. Sections were visualised using 3, 3′-diaminobenzidine (Sigma-Aldrich, St. Louis, MO, USA) and counterstained with Mayer’s haematoxylin.

### 4.5. TUNEL Assay

Terminal deoxynucleotidyl transferase dUTP nick end labelling (TUNEL) assay was performed using the In Situ Cell Death Detection Kit, Fluorescein (Roche, Basel, Switzerland) according to manufacturer instructions. Briefly, deparaffinised tissue sections were incubated for 8 min in freshly prepared permeabilization solution (0.1% Triton X-100, 0.1% sodium citrate) and washed with PBS. Subsequently, slides were incubated in the TUNEL reaction mixture containing terminal deoxynucleotidyl transferase and fluorescein dUTP labels in a dark humidified chamber for 60 min at 37 °C. For negative controls, reactions were performed without the labelling solution. For positive controls, slides were pre-treated with DNase I. For cover slipping, Vectashield mounting medium^TM^ with DAPI (Vector, Burlingame, CA, USA) was used, and samples were analysed under a fluorescence microscope (Olympus, Tokyo, Japan).

### 4.6. Analysis of Immune Cell Populations

Single cell suspensions from the mouse thymus, spleen, and mesenteric lymph nodes were prepared by mechanical disruption in complete RPMI 1640 without L-Glutamine (HyClone, Logan, UT, USA). Lymphocytes from perfused liver were isolated according to the method described by Graziani-Bowering and colleagues (1997) [53]. Red blood cells were removed by hypotonic lysis. Cells were then stained with FITC-conjugated anti-mouse antibodies specific for CD4, CD8, and TCRβ; PE-conjugated anti-mouse antibodies specific for CD8, CD11c, CD25, NK1.1, CD62L, CD69, CD44, and B220 (BD Biosciences, San Jose, CA, USA); and FITC-conjugated monoclonal antibodies specific for CD11b and Gr1 (eBioscience, San Diego, CA, USA). Stained cells were analysed using a FACSCalibur flow cytometer (Becton Dickinson, Mountain View, CA, USA). Two different channels were used for identifying cell type. FITC and PE signals were collected in FL1 through 530 nm and 585 nm bandpass filters, respectively. NKT cells were identified based on TCRβ^+^ and NK1.1^+^ signals. CD4^−^ and CD8^−^ positive cells were identified based on CD4^+^ and CD8^+^ signals, respectively. CD4^+^ and CD25^+^ signals represented regulatory T-cells. TCRβ^+^ cells and B220^+^ cells were assigned as T-cells and B-cells, respectively. For antigen-presenting cells, positive CD11b and CD11c signals indicated macrophages and dendritic cells, respectively. Granulocytes were recognised based on Gr1^−^ positive signals. To distinguish activated CD4^+^ and CD8^+^ cells, signals for CD44^+^, CD69^+^, and CD62L^−^ were additionally evaluated. Absolute cell numbers of each cell subset were calculated using the following formula: percentage of staining cells × total cell numbers.

### 4.7. Analysis of PG1 Cytotoxicity to Mammalian Cells

Expression constructs for pPG1 and mPG1 were designed, respectively, as described in Figure S13. Coding sequences of pre-pro and mature PG1 were amplified from pig PBMC cDNA via PCR using the primer sets pPG-F and –R, mPG-F (5′-ACT-CAG-ATC-TGG-TGG-CGG-TT-3′) and pPG-R. Amplicons were then subcloned into the multiple cloning site of the pEGFP-C1 mammalian expression vector (Clontech Laboratories, Inc., Mountain View, CA, USA). NIH3T3 (ATCC, Manassas, VA, USA) and 661W cells, which were shown to be derived from the cone photoreceptor cell lineage [54], were transfected with the plasmid construct using PolyMag™ transfection reagent (Chemicell, Berlin, Germany) following the manufacturer protocol. Briefly, cultured cells in 6-well culture dishes (SPL, Pocheon, South Korea) were incubated on the magnetic plate for 3 h with DMEM (HyClone, Logan, UT, USA) containing 6 µg of DNA and magnetic nanoparticle complexes. Then, transfected cells were continuously cultured without the magnetic plate for 16 and 24 h under 37 °C and 5% CO_2_ conditions. Cultured cells were suspended in 10 mM EDTA/PBS and filtered through a cell strainer cap (Becton Dickinson, Mountain View, CA, USA). The number of EGFP-positive cells was measured 16 and 24 h after transfection using a FACSCalibur™ flow cytometer (Becton Dickinson, Mountain View, CA, USA). EGFP signals were measured from the FL1 channel through a 515–545 nm bandpass filter. A scatter gate was drawn in the SSC versus FS plot to exclude debris and include the EGFP-positive cells using CellQuest™ software (Becton Dickinson, Mountain View, CA, USA).

### 4.8. Corneal Opacity Induction and Analysis

Synthesised mPG1 peptides were dissolved in PBS [16]. The eyes of wild-type C57BL/6 mice were treated with eye drops of PBS containing 12 µM of mPG1 every 12 h for 5 days. Three mice were used for the induction, and PBS alone was used for the control group. Corneal images were obtained under the common fluorescent light at three different time points, namely, before the treatment and 2 and 5 days after the treatment. The presence and absence of opacity were initially determined by visual observation and further analysed based on the pixel intensities of the corneal images. Regions of the same size showing opacity were analysed for each mouse using ImageJ software (NIH, Bethesda, MD, U.S).

### 4.9. Respiratory Infection and Post-Mortem Analysis

PG1 transgenic mice (*n* = 10) and wild-type littermates (*n* = 36) at 6 weeks old were used for experimental infection. They were maintained in isolator cages with a 12 h light/dark cycle and free access to food and water. After a week of acclimation period, the mice were anesthetized with diethyl ether and inoculated intranasally with *Staphylococcus aureus* (ATCC29213, 1 × 10^7^ CFU in 100 uL PBS). Mice received subcutaneous (s.c.) injections of nafcillin (100 mg/kg) or phosphate-buffered saline (PBS) every 4 h after infection. Mice were monitored for clinical signs every 4 h for 48 h post-inoculation as previously described [42]. Animals were euthanized at 4, 12, 24 and 48 h post-inoculation and the lungs were harvested and homogenized in PBS. All remaining mice were euthanized 48 h after inoculation. The guidelines for animal care and experimental protocols involving mice were described above.

### 4.10. Measurement of Bacterial Load

At necropsy, a half of the left lung lobes (about 45~50 mg) was dissected out and weighed in a sterile 1.5 mL tube. The 450 uL ice-cold PBS was added to the tube and tissues were immediately homogenized using an automated tissue homogenizer (Precelly^®^24; Bertin Technologies, France). Serial dilutions of lung tissue homogenates were plated onto blood agar plate (Hanil Komed, Seungnam, South Korea) and incubated overnight at 37 °C. Subsequently, the number of individual colonies was counted, and the titre was expressed as log10 CFU/g tissue. One-way analysis of variance (ANOVA) was used for statistical analysis.

### 4.11. Microscopic Evaluation of Lung Tissues

The right lung lobes were dissected out and fixed in 10% neutral buffered formaldehyde for 24 h. Tissues were cut into 5 μm thick sections using a microtome (Leica, Wetzlar, Germany) and mounted onto poly-l-lysine-coated glass slides. After deparaffinisation and hydration, sections were stained using haematoxylin and eosin (H&E) and analysed under a microscope (Olympus, Tokyo, Japan).

### 4.12. Cytokine Assay

The lung tissue was immediately homogenized at 4 °C using an automated tissue homogenizer. Lung homogenates were centrifuged at 900× *g* for 10 min at 4 °C. Supernatants were assessed for cytokine production using standard sandwich ELISA (eBioscience, San Diego, CA, USA).

## Figures and Tables

**Table 1:** Absolute numbers of immune cells in protegrin 1 (PG1) transgenic and wild-type mice.

Tissue	Population	Surface Phenotype	PG1 Transgenic	Wild-Type	*p*-Value
**Spleen (×10^5^)**	T cell	TCR-beta^+^	317.04 ± 22.46	305.65 ± 33.82	0.79
	CD4^+^ T cell	CD4^+^	216.46 ± 15.34	202.04 ± 25.42	0.64
	CD8^+^ T cell	CD8^+^	116.89 ± 8.04	122.96 ± 12.80	0.70
	Activated CD4^+^ T cell	CD4^+^CD44^+^	55.51 ± 3.33	43.93 ± 4.14	0.06
		CD4^+^CD69^+^	22.07 ± 2.47	21.40 ± 2.42	0.85
		CD4^+^CD62L-	57.39 ± 7.26	52.17 ± 6.92	0.61
	Activated CD8^+^ T cell	CD8^+^CD44^+^	27.94 ± 1.00	25.86 ± 3.04	0.54
		CD8^+^CD69^+^	4.04 ± 0.28	3.88 ± 0.24	0.67
		CD8^+^CD62L-	15.24 ± 3.76	10.31 ± 2.81	0.32
	Regulatory T cell	CD4^+^CD25^+^	23.23 ± 1.21	21.60 ± 2.28	0.55
	B cell	B220^+^	503.92 ± 36.70	466.04 ± 51.20	0.56
	Dendritic cell	CD11b^+^ Cd11c^+^	105.42 ± 10.62	95.17 ± 8.57	0.47
	Granulocyte	Gr1^+^ high	11.09 ± 4.31	13.01 ± 4.08	0.75
	Ly-6C^+^ monocytes	Gr1^+^low	71.49 ± 4.46	72.85 ± 8.12	0.89
	NK cell	NK1.1^+^ TCR-beta^+^	11.54 ± 0.58	11.54 ± 1.48	0.92
**Mesenteric Lymph node (×10^5^)**	T cell	TCR-beta^+^	87.71 ± 14.83	102.27 ± 9.12	0.43
	CD4^+^ T cell	CD4^+^	373.90 ± 26.00	351.61 ± 41.75	0.66
	CD8^+^ T cell	CD8^+^	250.44 ± 15.08	246.78 ± 23.92	0.90
	Activated CD4^+^ T cell	CD4^+^CD44^+^	7.48 ± 1.28	8.82 ± 0.61	0.37
		CD4^+^CD69^+^	9.23 ± 1.63	9.93 ± 0.95	0.72
		CD4^+^CD62L-	11.54 ± 2.31	12.30 ± 0.95	0.77
	Activated CD8^+^ T cell	CD8^+^CD44^+^	4.73 ± 0.90	6.40 ± 0.71	0.18
		CD8^+^CD69^+^	2.11 ± 0.43	2.54 ± 0.28	0.43
		CD8^+^CD62L-	4.73 ± 1.80	3.95 ± 0.42	0.69
	Regulatory T cell	CD4^+^CD25^+^	5.97 ± 1.20	6.39 ± 0.64	0.77
	B cell	B220^+^	36.34 ± 7.29	40.87 ± 4.86	0.62
	Dendritic cell	CD11b^+^ Cd11c^+^	5.50 ± 1.02	6.82 ± 1.06	0.39
	Granulocyte	Gr1^+^	0.08 ± 0.05	0.07 ± 0.03	0.88
	Ly-6C^+^ monocytes	Gr1^+^	9.51 ± 1.76	10.94 ± 1.79	0.58
**Thymus (×10^5^)**	CD4^+^ SP	CD4^+^	106.34 ± 11.57	85.44 ± 9.80	0.20
	CD8^+^ SP	CD8^+^	32.81 ± 2.57	26.78 ± 3.14	0.17
	DN	CD4-CD8-	35.27 ± 2.51	30.40 ± 3.26	0.27
	DP	CD4^+^CD8^+^	1033.98 ± 111.15	941.16 ± 162.80	0.65
	NK cell	NK1.1^+^ TCR-beta^+^	7.34 ± 0.69	6.36 ± 1.26	0.52
**Liver (×10^5^)**	NK cell	NK1.1^+^ TCR-beta^+^	0.27 ± 0.08	0.36 ± 0.08	0.38

Note. *p*-values were generated by unpaired, two-tailed Student’s *t* test. *n* = 6. All data represent the mean ± SEM. DN, double negative cell; DP, double positive; SP, single positive cell.

**Table 2:** Occurrence of corneal opacity in PG1 transgenic mice.

		Phenotypes	Total
	No. of Normal	No. of Corneal Opacity	
Total	161	54	215

Note: Phenotypes were determined at the age of 6 months.

## Supplementary Materials

The following are available online at https://www.mdpi.com/1422-0067/22/4/1586/s1, Figure S1. Comparison of the nucleotide sequences of the upstream region of the MUC1 start codon between mice and pigs. mMuc1 and pMUC1 represent the promoter regions of mouse and pig mucin 1 genes, respectively. The green and blue shades indicate the TATA box and start codon, respectively. Figure S2. Vector map of the pMUCPG1 transgenic construct. The pPG1 coding sequence was inserted between the *XbaI* and *Xho*I restriction sites. Figure S3. Screening of PG1 transgenic mice using PCR. Primers, pPG1-F and –R, were used to determine the insertion of *PG1* into the genome. The 465-bp of *PG1* specific amplicons were obtained from PG1 transgenic mice. M, marker; TG, PG1 transgenic; wild, wild-type (C57BL/6); +, positive control (PG1 construct); -, negative control. Figure S4. Tissue distribution of PG1 expression in PG1 transgenic mice from semi-quantitative RT-PCR. A representative image of the gel pictures (*n* = 3) from the semi-quantitative reverse-transcription PCR. (A) Fourteen different tissues were analysed for the expression of PG1 transcripts. *PG1*-specific amplicons (293 bp) were amplified using the primers PGRT-F and PGRT-R. The expression level was represented as a bar plot of pixel intensity ratio (*PG1/GAPDH*). Lanes 1. neocortex, 2. eye, 3. lung, 4. thymus, 5. liver, 6. kidney, 7. stomach, 8. small intestine, 9. spleen, 10. ovary, 11. uterus, 12. testis, 13. muscle, and 14. skin. (B) The expression of PG1 in peripheral blood mononuclear cells (PBMCs) of PG1 transgenic mice. Figure S5. Results of immunohistochemical analysis using anti-PG1 antibodies in the retina, lung, intestine and stomach of MUC1-PG1 transgenic mice. Synaptic structures in the outer plexiform layer of three-month-old wild-type mouse (C57BL/6, A) and PG1 transgenic mouse (B) were stained with anti-PG1 antibodies and are indicated with arrows (original objective 60×). However, the retinal abnormality was not detected in PG1 transgenic mice at 4 weeks old. In the lung (C, original objective 40 ×), PG1 positive cells were identified along the alveolar sacs. Cell types were indicated with arrows. Strong PG1 positive signals were shown at the mucosal layers of stomach (D, original objective 40×). Figure S6. Representative images of the appearance of lungs from different treatment groups after infection with *Staphylococcus aureus*. Patchy haemorrhage appeared in infected lungs beginning from 12 h after infection. The number of mice for each time point was indicated within parentheses. The haemorrhage appears more severe in the tissue of infected wild-type mice compared to other groups. WT, wild-type; Tg, PG1 transgenic; Group 4, negative group. Figure S7. Histological observation of lung tissue sections. Samples from different treatment groups were analysed 48 h after infection with *Staphylococcus aureus*. Groups 1, 2, 3, 4, and 5 correspond to WT infected, Tg infected, WT infected plus nafcillin treatment, and WT not infected, Tg not infected, respectively. WT, wild-type; Tg, PG1 transgenic. Representative images of tissue sections from H&E staining. There are varying degrees of acute congestion, collapse of the alveolar lumen and neutrophilic infiltration among different groups and indicated by arrows. The rectangular insets in the 40x images indicate regions with pathological abnormalities and were magnified in 100x resolution. Figure S8. Comparison of bacterial cell counts from the lung after intranasal infection of *Staphylococcus aureus* between transgenic and wild-type mice. The numbers represent bacterial colony forming units (CFUs) from lung homogenates between wild-type and PG1 transgenic mice after 4, 12, 24 and 48 h after infection (*, *p* < 0.05 and **, *p* < 0.01). Data represent the means ± SEMs. WT, wild-type; Tg, transgenic; Group 4, control group. Figure S9. Comparison of macrophage and monocyte infiltration in lung tissue sections between wild-type and PG1 transgenic mice. Samples from different treatment groups were analysed 48 h after infection with *Staphylococcus aureus*. Groups 1, 2, 3, and 4 correspond to WT infected, Tg infected, WT infected plus nafcillin treatment, and WT not infected, respectively. WT, wild-type; Tg, PG1 transgenic. Representative images of tissue sections from immunohistochemical analysis using anti-macrophage/monocyte antibodies were shown. Antibody specific signals appear in brown and indicated by arrows. Figure S10. Representative images of corneal opacity-affected eyes and subsequent eye deformation in PG1 transgenic mice. The right eye of a female PG1 transgenic mouse at the age of 4 weeks showing pronounced early-onset corneal opacity (A). Eyes of PG1 transgenic mice of the same age (three-month-old) at different stages of abnormalities (B). Figure S11. A representative picture of the inflammation in eye lids in MUC1-PG1 transgenic mice. Haematoxylin and eosin Y staining of eyelid and ocular regions in wild-type mice (A,C,E) and transgenic mice (B,D,F,G,H). Affected transgenic mice showed thicker eyelid epithelia compared to wild-type mice. Additionally, affected mice showed a significantly higher number of infiltrated granulocyte-like cells in the eyelid and conjunctival regions (C–H) compared to wild-types. Representative images of H&E staining from intermediately (G) or severely (H) progressed abnormalities. Arrows indicate multi nucleus or eosinophilic cells. The symbol “**” in figure F also indicates a cluster of infiltrated granulocyte-like cells. (A,B): 100×, (C,D,E,F): 400×, (G,H): 40×. Figure S12. Corneal opacity in wild-type mice induced by PG1 treatment. Eyes of wild-type mice were treated with an eye drop solution containing synthesised PG1 every 12 h for 5 days. (A) Representative corneal images of animals treated with phosphate buffered saline (PBS) or mature PG1 peptides after 0, 2, and 5 days (A top). For better visualisation, the colours of corneal images were inverted (A bottom). (B) Results of opacity analysis. Pixel intensity within the same-sized circle in the original images were measured using ImageJ software. Bars indicate the difference in pixel intensity for corneal region of PBS (1–3) and PG1-treated animals (4–6) at after 2 and 5 days of treatment. Error bars indicate standard deviation from three different photos of the single animal. Three animals were tested per group. Figure S13. Vector map of *EGFP-PG1* transfection construct. pPG1 (A) and mPG1 (B) were inserted into *Bgl*II and *Xho*I restriction sites, respectively, to produce EGFP fusion proteins. pEGFP-C1 was used as a backbone vector. Figure S14. Immunohistochemical analysis of the Harderian gland of MUC1-PG1 transgenic mice using anti-ELANE antibodies. Results of anti-ELANE immunohistochemistry for the Harderian gland. Antibody reactivity is indicated in brown by diaminobenzidine (DAB) staining. Type-I cell-like cells in Harderian glands (600×) showed positive signals for ELANE. (i) lumen, (ii) columnar cells with basal nuclei and vacuoles. Table S1. Cell counts in PG1 transgenic and wild-type mice Table S2. Bacterial challenge experimental design.
