# Supplementary material for: Transgenic Mice Overexpressing PG1 Display Corneal Opacity and Severe Inflammation in the Eye
Source: Int J Mol Sci. 2021 Feb 4;22(4):1586. doi: 10.3390/ijms22041586 (PMC7915784; doi:10.3390/ijms22041586)
Supplement: Supplementary file 1 [file ijms-22-01586-s001.pdf]

Supplementary data

Transgenic mice overexpressing PG1 display corneal opacity and severe inflammation in t he eye
Min-Kyeung Choi[1], Minh Thong Le[1], Hyesun Cho[1], Juyoung Lee[1], Hyoim Jeon[1], Se-Yeoun Cha[2], Manhe um Na[3], Taehoon Chun[3], Jin-Hoi Kim[1], Hyuk Song[1], Chankyu Park[1]*

1Department of Stem Cell Biology and Regenerative Biology, Konkuk University, Hwayang-dong, Seoul, Republic of Korea; 2College of Veterinary Medicine, Chonbuk National University, Iksan, Republic of K orea; 3Department of Biotechnology, College of Life Sciences and Biotechnology, Korea University, Seo ul, Republic of Korea

Seoul 143-701, Korea
Tel: 82-2-450-3697; fax: 82-2-457-8488
E-mail: chankyu@konkuk.ac.kr

- Figure S1. Comparison of the nucleotide sequences of the upstream region of the MUC1 start codon between mice and pigs.
- Figure S2. Vector map of the pMUCPG1 transgenic construct.
- Figure S3. Screening of PG1 transgenic mice using PCR.
- Figure S4. Tissue distribution of *PG1* expression in PG1 transgenic mice from semi-quantitative RT -PCR.
- Figure S5. Results of immunohistochemical analysis using anti-PG1 antibodies in the retina, lung, inte stine and stomach of MUC1-PG1 transgenic mice.
- Figure S6. Representative images of the appearance of lungs from different treatment groups after in fection with *Staphylococcus aureus*.
- Figure S7. Histological observation of lung tissue sections
- Figure S8. Comparison of bacterial cell counts from the lung after intranasal infection of *Staphyloco ccus aureus* between transgenic and wildtype mice.
- Figure S9. Comparison of macrophage and monocyte infiltration in lung tissue sections between wildtype and PG1 transgenic mice.
- Figure S10. Representative images of corneal opacity-affected eyes and subsequent eye deformation i n PG1 transgenic mice.
- Figure S11. Corneal opacity in wild-type mice induced by PG1 treatment.
- Figure S12. Vector map of EGFP-PG1 transfection construct.
- Figure S13. Immunohistochemical analysis of the Harderian gland of MUC1-PG1 transgenic mice usi ng anti-ELANE antibodies.
- Figure S14. Analysis of ELANE expression using semi-quantitative RT-PCR.
- Table S1. Cell counts in PG1 transgenic and wild type mice
- Table S2. Bacterial challenge experimental design

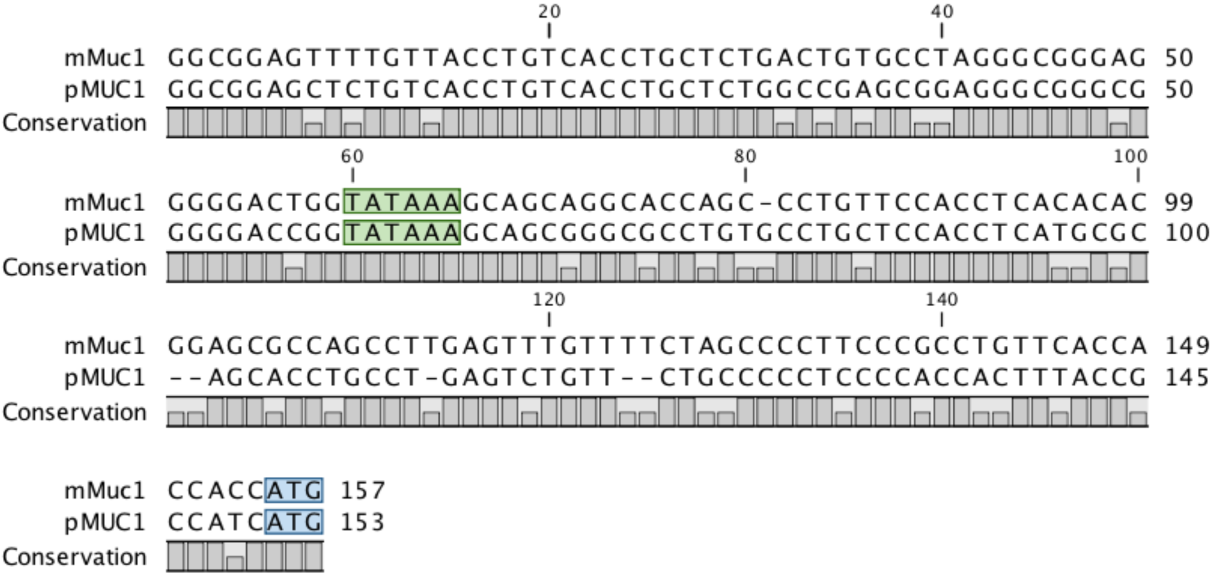

Figure S1. Comparison of the nucleotide sequences of the upstream region of the MUC1 start codon between mice and pigs. *mMuc1* and *pMUC1* represent the promoter regions of mouse and pig mucin 1 genes, respectively. The green and blue shades indicate the TATA box and start codon, respectively.

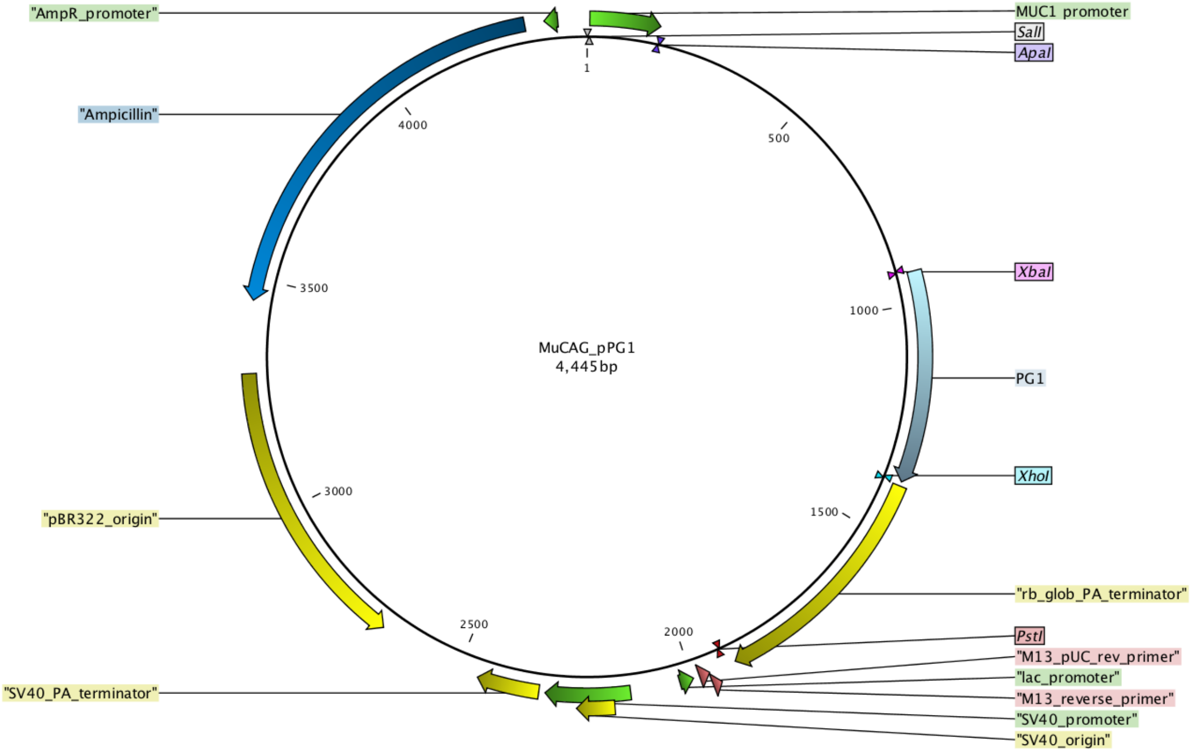

Figure S2. Vector map of the pMUCPG1 transgenic construct. The pPG1 coding sequence was inserted between the *XbaI* and *XhoI* restriction sites.

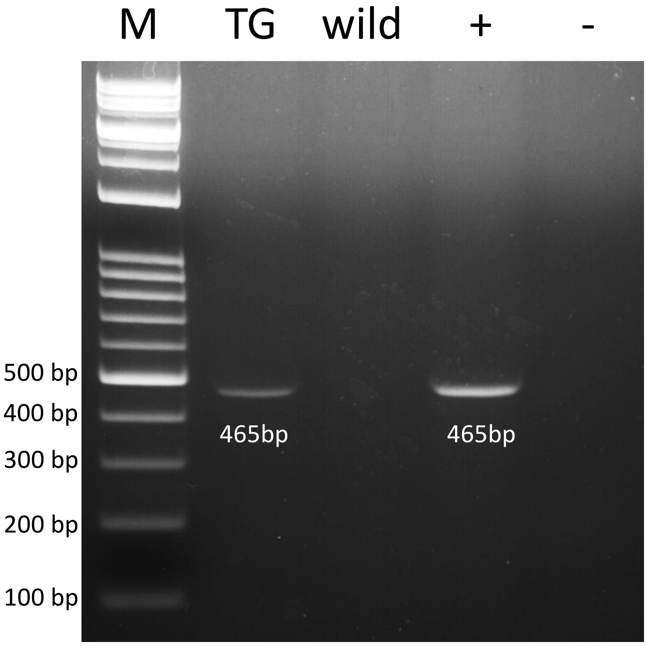

Figure S3. Screening of PG1 transgenic mice using PCR. Primers**,** pPG1-F and –R, were used to determine the insertion of PG1 into the genome. The 465 bp of PG1 specific amplicons were obtained from PG1 transgenic mice. M, marker; TG, PG1 transgenic; wild, wild type (C57BL/6); +, positive control (PG1 construct); -, negative control.

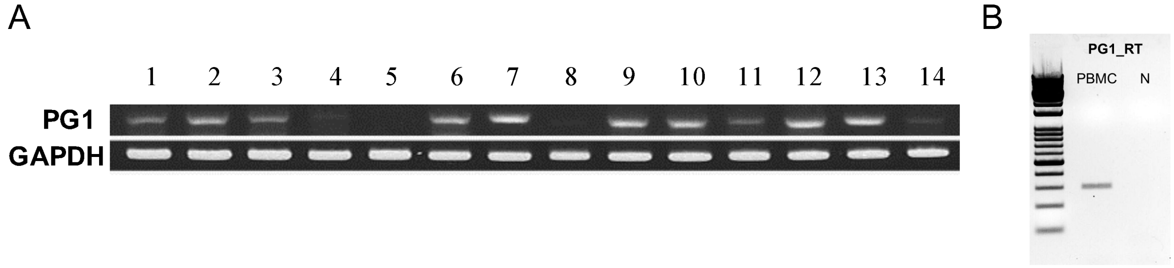

Figure S4. Tissue distribution of *PG1* expression in PG1 transgenic mice from semi-quantitative RT-PCR. A representative image of the gel pictures (n=3) from the semi-qua ntitative reverse-transcription PCR. (A) Fourteen different tissues were analysed for the e xpression of *PG1* transcripts. PG1-specific amplicons (293 bp) were amplified using the p rimers PGRT-F and PGRT-R. The expression level was represented as a bar plot of pixel in tensity ratio (*PG1/GAPDH*). Lanes 1. neocortex, 2. eye, 3. lung, 4. thymus, 5. liver, 6. kid ney, 7. stomach, 8. small intestine, 9. spleen, 10. ovary, 11. uterus, 12. testis, 13. muscle, and 14. skin. (B) The expression of *PG1* in peripheral blood mononuclear cells (PBMC) of PG1 transgenic mice.

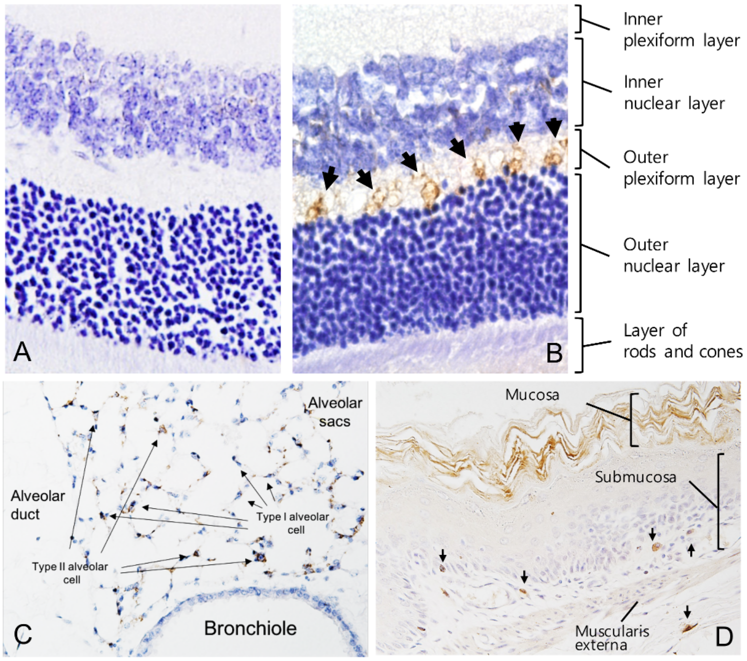

Figure S5. Results of immunohistochemical analysis using anti-PG1 antibodies in th e retina, lung, intestine and stomach of MUC1-PG1 transgenic mice. Synaptic struct ures in the outer plexiform layer of three-month-old wild type mouse (C57BL/6, A) and PG1 transgenic mouse (B) were stained with anti-PG1 antibodies and are indicated with arrows (original objective 60 x). However, the retinal abnormality was not detected in P G1 transgenic mice at 4 weeks old. In the lung (C, original objective 40 x), PG1 positive cells were identified along the alveolar sacs. Cell types were indicated with arrows. Stro ng PG1 positive signals were shown at the mucosal layers of stomach (D, original object ive 40 x).

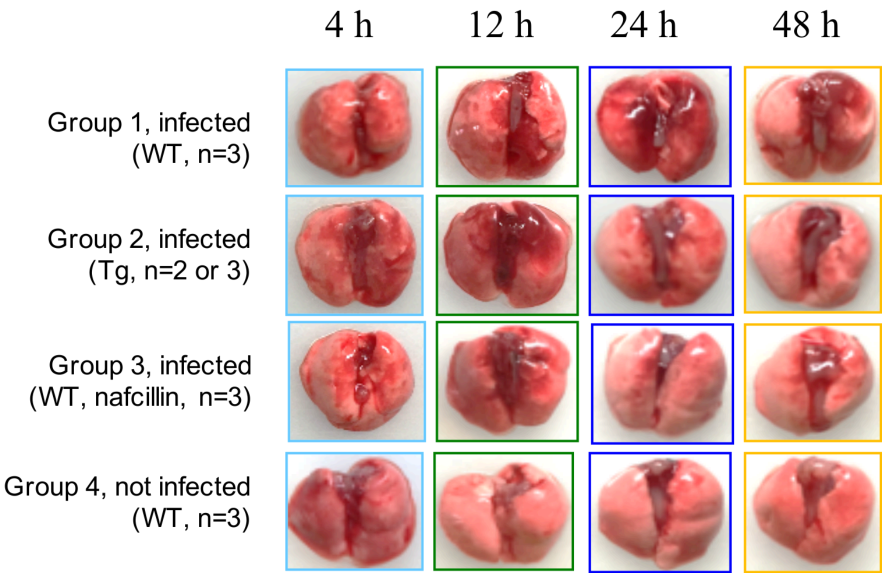

Figure S6. Representative images of the appearance of lungs from different treatment groups after infection with *Staphylococcus aureus*. Patchy hemorrhage appeared in infected lungs beginning from 12 h after infection. The number of mice for each time points was indicated within parentheses. The hemorrhage appears more severe in the tissue of infected wildtype mice comparing to other groups. WT, wildtype; Tg, PG1 transgenic; Group 4, negative group.

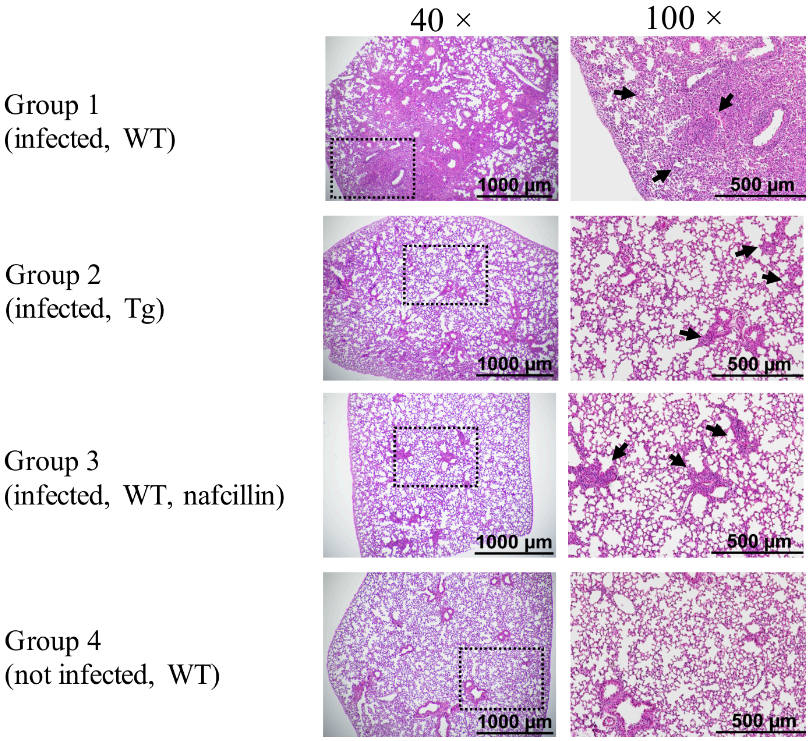

Figure S7. Histological observation of lung tissue sections. Samples from different treatment groups were analysed 48 h after infection with *Staphylococcus aureus.* Groups 1, 2, 3, and 4 correspond to WT infected, Tg infected, WT infected plus nafcillin treatment, and WT not infected, respectively. WT, wildtype; Tg, PG1 transgenic. (A) Representative images of tissue sections from H&E staining. There are varying degrees of acute congestion, collapse of the alveolar lumen and neutrophilic infiltration among different groups and indicated by arrows. The rectangular insets in the 40x images indicate regions with pathological abnormalities and were magnified in 100x resolution.

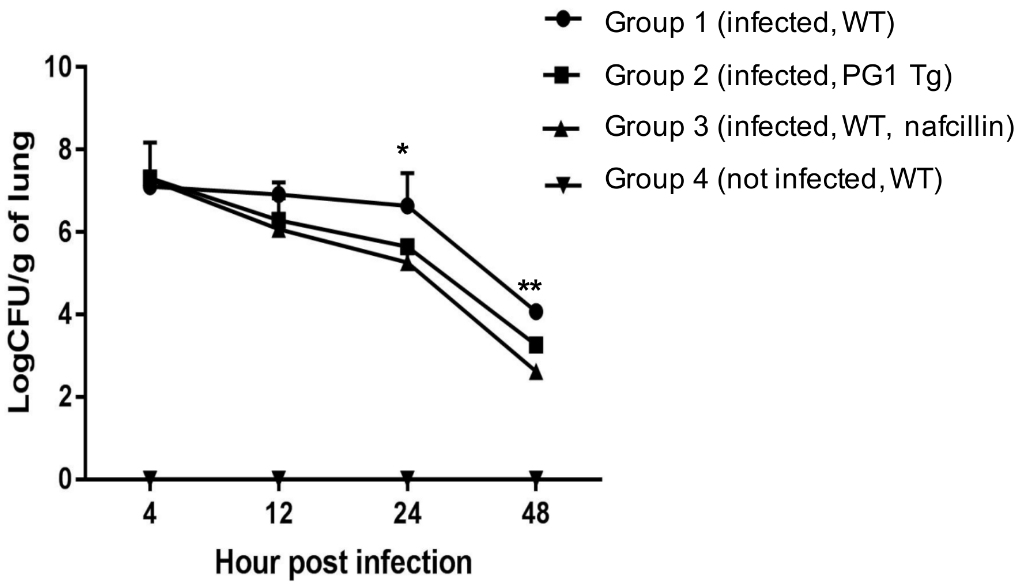

Figure S8. Comparison of bacterial cell counts from the lung after intranasal infection of *Staphylococcus aureus* between transgenic and wildtype mice. The numbers represent bacterial colony forming units (CFUs) from lung homogenates between wildtype and PG1 transgenic mice after 4, 12, 24 and 48 h after infection (*, $P<0.05$ and **, $P<0.01$). Data represent the means ± SEMs. WT, wildtype; Tg, transgenic; Group 4, control group.

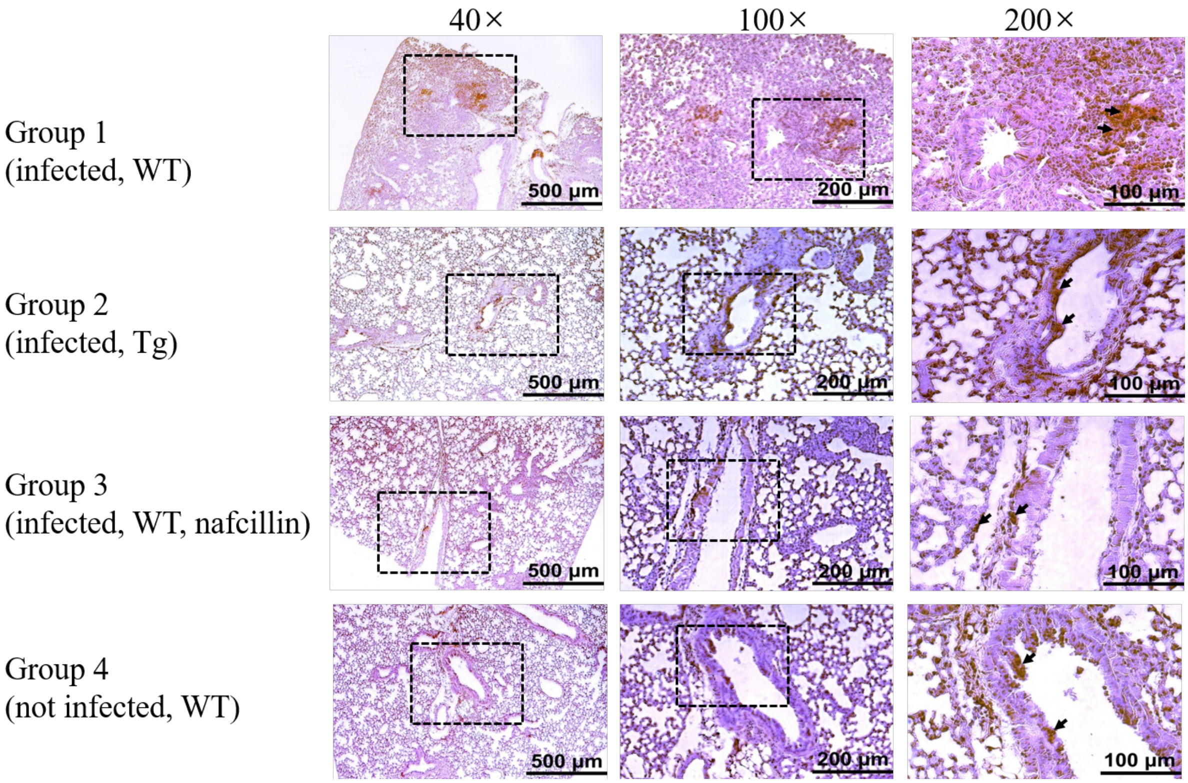

Figure S9. Comparison of macrophage and monocyte infiltration in lung tissue sections between wildtype and PG1 transgenic mice. Samples from different treatment groups were analysed 48 h after infection with *Staphylococcus aureus.* Groups 1, 2, 3, and 4 correspond to WT infected, Tg infected, WT infected plus nafcillin treatment, and WT not infected, respectively. WT, wildtype; Tg, PG1 transgenic. Representative images of tissue sections from immunohistochemical analysis using anti-macrophage/monocyte antibodies were shown. Antibody specific signals appear in brown and indicated by arrows.

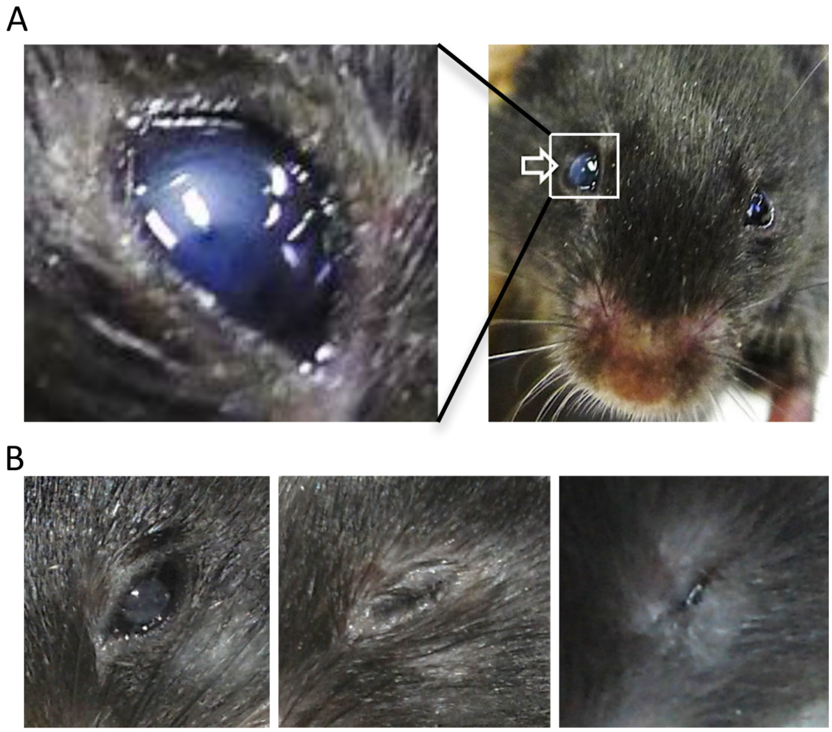

Figure S10. Representative images of corneal opacity-affected eyes and subsequent eye deformation in PG1 transgenic mice. The right eye of a female PG1 transgenic mouse at the age of 4 weeks showing pronounced early-onset corneal opacity (A). Eyes of PG1 transgenic mice of the same age (three-month-old) at different stages of abnormalities (B).

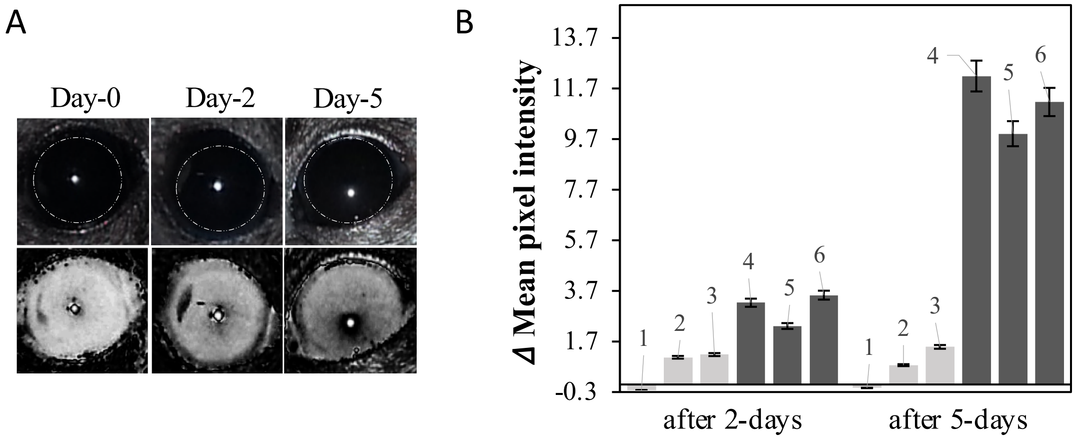

Figure S11. Corneal opacity in wild-type mice induced by PG1 treatment. Eyes of wild-type mice were treated with an eye drop solution containing synthesised PG1 every 12 h for 5 days. A) Representative corneal images of animals treated with phosphate buffered saline (PBS) or mature PG1 peptides after 0, 2, and 5 days (A top). For better visualisation, the colours of corneal images were inverted (A bottom). B) Results of opacity analysis. Pixel intensity within the same-sized circle in the original images were measured using ImageJ software. Bars indicate the difference in pixel intensity for corneal region of PBS (1-3) and PG1-treated animals (4-6) at after 2 and 5 days of treatment. Error bars indicate standard deviation from three different photos of the single animal. Three animals were tested per group.

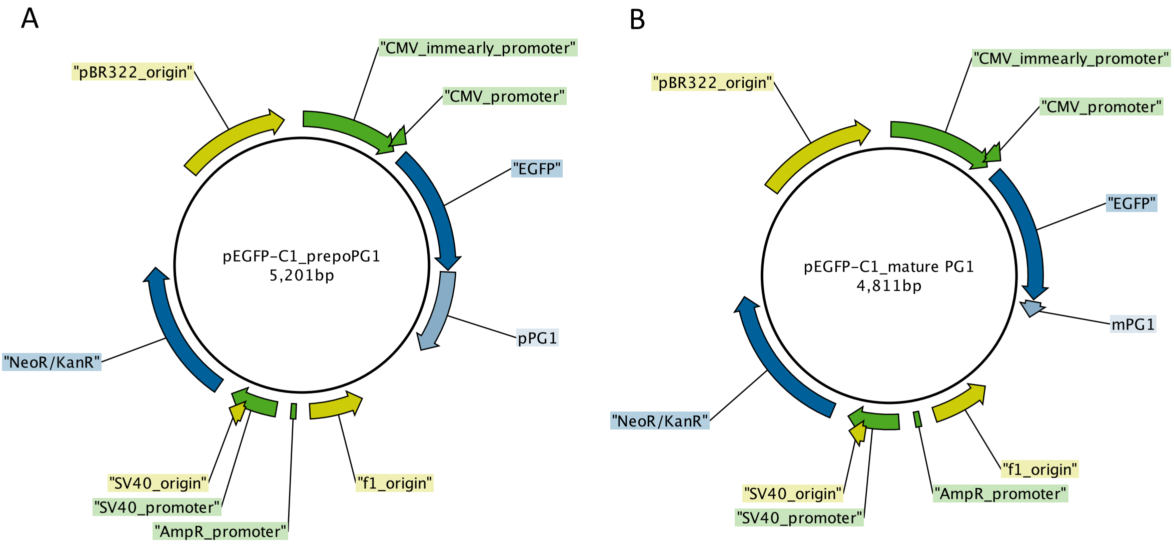

Figure S12. Vector map of EGFP-PG1 transfection construct. pPG1 (A) and mPG1 (B) were inserted into *BglII* and *XhoI* restriction sites, respectively, to produce EGFP fusion proteins. pEGFP-C1 was used as a backbone vector.

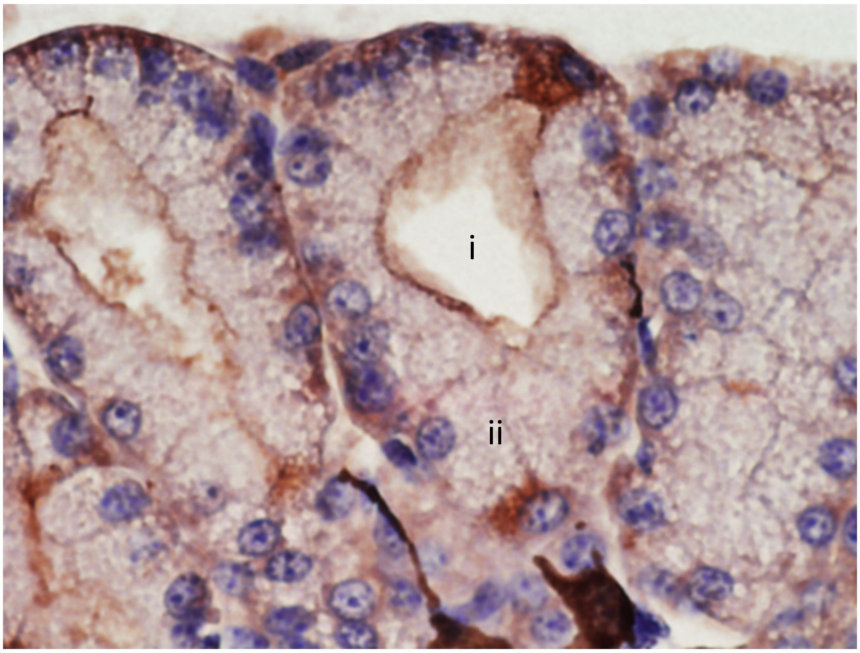

Figure S13. Immunohistochemical analysis of the Harderian gland of MUC1-PG1 transgenic mice using anti-ELANE antibodies. Results of anti-ELANE immunohistochemistry for the Harderian gland. Antibody reactivity is indicated in brown by diaminobenzidine (DAB) staining. Type-I cell-like cells in Harderian glands (600x) showed positive signals for ELANE. i) lumen, ii) columnar cells with basal nuclei and vacuoles.

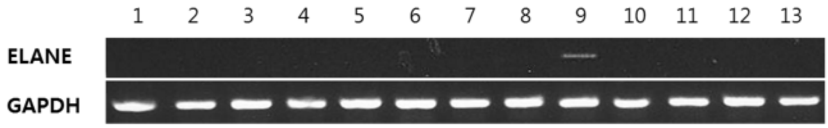

Figure S14. Analysis of *ELANE* expression using semi-quantitative RT-PCR. Thirteen tissues were analysed. Results were identical between transgenic and wild-type mice. Lanes 1. neocortex, 2. eye, 3. thymus, 4. lung, 5. stomach, 6. small intestine, 7. liver, 8. kidney, 9. spleen, 10. testis, 11. seminal vesicle, 12. muscle, 13. skin. *GAPDH* was used as internal control.

Table S1. Cell counts in PG1 transgenic and wild type mice

Tissue	PG1 transgenic	Wild type	p-value
Thymus (x10^7)	12.54 ± 1.23	11.24 ± 1.78	0.56
Spleen (x10^7)	9.93 ± 0.73	9.27 ± 0.97	0.60
Mesenteric Lymph node (x10^7)	1.39 ± 0.24	1.60 ± 0.15	0.48
Liver (x10^5)	9.13 ± 2.10	8.51 ± 1.80	0.83

Table S2. Bacterial challenge experimental design

<table>
<tr><th>Group</th><th>Mice</th><th>Age</th><th>No.</th><th>Strain</th><th>Challenge Dose and volume</th><th>Antibiotic concentration</th><th>Route</th><th>Time</th><th>Evaluation</th></tr>
<tr><td>1</td><td>Non-transgenic mouse C57BL/6</td><td rowspan="4">6 w</td><td>12</td><td rowspan="3">S. aureus ATCC29213</td><td rowspan="3">LD_0</td><td>-</td><td rowspan="4">Intranasal</td><td rowspan="4">2 day</td><td rowspan="4">1. Clinical signs (including body weight) (every 4 hour)
2. Gross lesions (4, 12, 24, 48h)
3.Bacterial load (lung, blood) (4, 12, 24, 48h)
4. Histopathology (48h)
5.Cytokine assays (48h) (TNF-α and IL-6)</td></tr>
<tr><td>2</td><td>Non-transgenic mouse C57BL/6</td><td>12</td><td>Nafcillin 100mg/kg</td></tr>
<tr><td>3</td><td>Transgenic mouse C57BL/6</td><td>10</td><td>-</td></tr>
<tr><td>4</td><td>Non-transgenic mouse C57BL/6</td><td>12</td><td>PBS</td><td>100μL</td><td>-</td></tr>
</table>

No. : Number of animals
